# Micrometers

**Published:** 1885-07

**Authors:** D. R. Ford


### MICROMETERS.

PROF. D. R. FORD.

In nothing is human judgment more at fault than as to the size of things. "As big as an elephant, or as diminutive as a grain of sand, were long ago considered accurate enough definitions." But now it is different. Elephants vary in size; so do grains of sand. Things must be measured nowadays; no guess work allowed. "How large is the sun?" "As big as a cart wheel," says Labienus, lieutenant of Julius Cæsar. "Not so," answers Sir John Herschel, officer of Victoria; "it is 888,812 miles in diameter, subject to rectification by better parallax."

With the advance of recent science, fine, or vast but always accurate, measurements are necessarily employed. Guess work, slack joints, and rule o' thumb, are no longer allowed. Astronomy owes her latest triumphs to improved telescopes and micrometers as much as to triangles and logarithms. In some departments of mechanic arts, the tight joints made one hundred years ago are now repudiated as too open and coarse. Accurate gauges are required.

Chemistry is greatly indebted to more accurate weighing. She aims to weigh molecules and atoms. I cannot prove or disprove the inference of two eminent chemists, that the weight of a hydrogen atom is one microcrith; a weight which is one-half of one octillionth, of nine one-hundredths of a gram, very nearly. True or not, this inference deals with ultimate littleness, and must be gratifying as a sort of standard for weighing our ponderous bacteria with.

Turning now from weights to linear measures, we find some rather astonishing claims set up. Nobert, of Vienna, claims to have ruled with a diamond point on plate glass 1,000,000 parallel grooves in a space of one inch. Fasoldt, of Albany, I hear, can do the same. No man ever yet saw these one-millionth of an inch spaces between the ruled lines, but from the proportional parts of the ruling engines which made these micrometers, the highest authorities agree that it is probable. The optical effects, also, of Nobert's lines go to establish his claim. I see no reason to doubt these wonderful rulings so far beyond the power of any microscope, yet to resolve.

While we are at it, let us turn to wonderful things in square measure. Take a square inch of paper and its size is comprehensible. But Mr. Webb, a microscopist of London, has made a machine called a micropantagraph, which he uses for executing extremely minute writing and engraving. With this instrument he engraved on a slip of glass, the Lord's prayer consisting of 227 letters in the space of $\frac{1}{129654}$ of a square inch. This is at the rate of 29,431,458 letters to the inch, which is rather more than eight complete Bibles; the common version on a careful count being found to contain 3,566,480 letters. The area within which the Prayer was written was micrometrically verified by Dr. J. J. Woodward, of the United States Medical Army Museum, at Washington, a very high authority in such matters. The success of Webb and Nobert in these achievements is probably due to the extreme minuteness of the points they employ whether they are diamond or sapphire. The secret, at any rate, has been well kept.

Prof. Rogers, of Cambridge, who has executed some admirable micrometric rulings with an engine of his own invention, seems to think as I understood him, that the most choice point is that of a natural diamond crystal. Out of a large number of minute diamond points, the operator will at length find one by trying that will do the work. Such a point once found is a treasure. He has just sent me (a generous gift from our Senior class) one of Prof. Rutherford's diffraction plates, containing these minute parallel rulings upon speculum metal, having 17,290 lines to the inch. It is to be used for securing spectra of the sun spots and solar flames.

Micrometers are used on telescopes, for measuring small distances, and apparent diameters of bodies which subtend minute angles. On microscopes they are used to measure direct distances which are exceeding small. There are many kinds in use. I name a few as follows; Dioptric micrometer, photographic micrometer, ring micrometer, divided object glass micrometer, Filar micrometer, double refraction micrometer, stage micrometer, ocular, etc., etc.

Let us assume, in learning the use of a micrometer with a microscope, that we have one of these fine rulings on glass, say 1000 lines to the inch. We lay it upon the stage, and lay the object to be measured directly upon it. Suppose its greatest length just covers 10 spaces between the rulings. It is evident your object is $\frac{10}{1000}$ or $\frac{1}{100}$ of an inch in length. So try any other diameter. If your rulings are accurately made, you have a confident measure. For verifying their accuracy we may; 1st compare them with some object of well tested diameter; or 2d, get them verified by one or more rival ruling engines; or 3d, measure the same object by micrometers of different makers of good repute, and take the mean value of them all; or 4th, measure the same object by the different bands of ruling on the same micrometer, say with the 1,000 band, with the 2,500 band, to observe if the measures are consistent with each other; 5th, or get a micrometer from some maker of the highest repute, then in your record of measurements always state the date and maker of your micrometer. You can thus be able to make allowances afterward if you ever find a more accurate one.

Whoever uses a stage micrometer, however good, will have much trouble with holding diatoms and other small objects steadily in the same place. They will slide around or fall off. There are other difficulties almost as bad. On the whole then we may say that a stage micrometer makes slow and often bungling work. These difficulties have given rise to the invention of several eyepiece or ocular micrometers. It is thought by some, that the Filar or cobweb micrometer is the best. It is much used for fine work with telescopes. But to be uniform, we will select one of a different sort, say a millimetre micrometer, or a fractional inch one. It will be a slip of fine, thin glass about an inch long and a third of an inch wide, upon which excessively fine parallel rulings have been accurately made. This is to be fitted to the eyepiece across it between the two lenses. By looking we shall see its rulings projected apparently upon the stage of the microscope. They will seem wider apart the higher objective we use. Suppose we try it with a one inch eyepiece and a half inch objective, to get the value of each space between the lines or rulings. We must place a good stage micrometer with say 1,000 lines to the inch, upon the stage and carefully observe how many of the spaces of the eyepiece micrometer exactly fill one space of our well known stage micrometer. Suppose it takes three, then obviously the value of each space in the eyepiece micrometer is equal to one-third of one-thousandth of an inch or one three-thousandths of an inch. Now we next put on the microscope a $\frac{1}{4}$ inch objective, and find the values with this new magnification in the same way. Find the values of the eyepiece spaces for all your objectives, and have them recorded in a handy place in your microscope box for use. Of course if you are rich in eyepieces and wish to use this convenient micrometer with each of them, it will be necessary to determine and record the values of your eyepiece micrometer with each ocular, as well as objective; in short. with each fresh combination of ocular and objective. Suppose we go back now and measure a given diatom with our one inch ocular and $\frac{1}{2}$ inch objective. We place the glass slide and diatom upon the stage, and focus properly. All that remains is to observe and count carefully the number of rulings it takes for the diameter of the diatom, blood corpuscle, pollen granule, or what not, and if it be two, then it must be $\frac{2}{3000}$ of an inch, or $\frac{1}{1500}$ of an inch, and so on.

—THE FIRST ingredient in conversation is truth, the next good sense, the third good humor, and the fourth wit.

